# Learning Curve in a Western Training Center of the Circumferential En Bloc Esophageal Endoscopic Submucosal Dissection in an In Vivo Animal Model

**DOI:** 10.1155/2011/847831

**Published:** 2011-10-03

**Authors:** Miguel A. Tanimoto, Gonzalo Torres-Villalobos, Rikiya Fujita, Patricio Santillan-Doherty, Jorge Albores-Saavedra, Fredy Chable-Montero, Luis A. Martin-del-Campo, Lucia Vasquez, Carlos Bravo-Reyna, Octavio Villanueva, Jose J. Villalobos, Misael Uribe, Miguel A. Valdovinos

**Affiliations:** ^1^Gastroenterology Department, World Gastroenterology Organisation Training Center, Instituto Nacional de Ciencias Medicas y Nutricion Salvador Zubiran, Vasco de Quiroga No. 15, Colonia Seccion XVI, Delegacion Tlalpan, 14000 Mexico City, DF, Mexico; ^2^Surgery and Experimental Surgery Department, Instituto Nacional de Ciencias Medicas y Nutricion Salvador Zubiran, Vasco de Quiroga No. 15, Colonia Seccion XVI, Delegacion Tlalpan, 14000 Mexico City, DF, Mexico; ^3^Shinmidori Hospital Yokohama, Yokohama 226-0025, Japan; ^4^Pathology Department, Instituto Nacional de Ciencias Medicas y Nutricion Salvador Zubiran, Vasco de Quiroga No. 15, Colonia Seccion XVI, Delegacion Tlalpan, 14000 Mexico City, DF, Mexico; ^5^Experimental Surgery Department, Instituto Nacional de Ciencias Medicas y Nutricion Salvador Zubiran, Mexico City, Mexico; ^6^Animal Lab and Experimental Research Department, Instituto Nacional de Ciencias Medicas y Nutricion Salvador Zubiran, Vasco de Quiroga No. 15, Colonia Seccion XVI, Delegacion Tlalpan, 14000 Mexico City, DF, Mexico

## Abstract

*Aim*. Evaluate the feasibility to overcome the learning curve in a western training center of the en bloc circumferential esophageal (ECE-) ESD in an in vivo animal model. *Methods*. ECE-ESD was performed on ten canine models under general anesthesia on artificial lesions at the esophagus marked with coagulation points. After the ESD each canine model was euthanized and surgical resection of the esophagus and stomach was carried out according to “the Principles of Humane Experimental Technique, Russel and Burch.” The specimen was fixed with needles on cork submerged in formalin with the esophagus and stomach then delivered to the pathology department to be analyzed. *Results*. ECE-ESD was completed without complications in the last 3/10 animal models. Mean duration for the procedures was 192 ± 35 minutes (range 140–235 minutes). All the procedures were done at the animal lab surgery room with cardio pulmonary monitoring and artificial ventilation by staff surgery members and a staff member of the Gastroenterology department trained during 1999–2001 at the Fujigaoka hospital of the Showa U. in Yokohama, Japan, length (range 15–18 mm) and 51 ± 6.99 width (range 40–60 mm). *Conclusion*. ECE-ESD training is feasible in canine models for postgraduate endoscopy fellows.

## 1. Introduction

The incidence of esophageal adenocarcinoma is currently rising in Western countries and Latin America. Advanced cancer in the esophagus is a serious and lethal disease that extends locally to deeper layers of the esophageal wall with significant risk of nodal metastasis and invasion of adjacent organs. The appropriate management of patients in whom early carcinoma or HGIN (high-grade intraepithelial neoplasia) has been detected continues to be a subject of controversy in the Western countries and Latin America [1]. For many years, radical esophageal resection has been regarded as the treatment of choice, but because of the morbidity and mortality rates associated with the procedure, less radical treatment strategies have been advocated by many groups. Other therapeutic approaches include ablative treatment methods, all of which have in common that they do not provide a viable specimen of the neoplastic lesion for exact histological confirmation, infiltration of the depth, and presence of invasion of lymph vessels (L-status) or blood vessels (V-status) [2]. With these available ablative treatment modalities, a possible problem might be the underestimation of a neoplastic lesion and the endoscopist might end up treating a submucosal carcinoma or an infiltrating cancer with lymphatic spread with a risk of undertreatment of an undetected submucosal cancer, a finding which occurs in 12.7% of patients with HGIN. This risk seems to be even higher when ablation of HGIN is performed in less experienced centers. This hypothesis is underlined by the fact that most HGIN and early invasive cancers are found in flat mucosa. One reliable method of avoiding this is to detect lesions at an early stage of esophageal cancer and resect them locally [3]. Endoscopic resection is a lower-risk procedure alternative to surgery for the management of HGIN and intramucosal cancer. In Western countries currently it is performed mainly using EMRL (endoscopic mucosal resection using cap and band ligation), a piecemeal endoscopic resection technique which has been widely accepted for the treatment of early Barrett's neoplasia with the inconvenience of their recurrence rates [4]. 

 In contrast en-bloc esophageal ESD (endoscopic submucosal dissection) is only performed in Asian countries for several reasons. It is a highly skilled procedure that requires tutorial training in order to overcome the learning curve. On the other hand circumferential en-bloc esophageal ESD is even more challenging and is considered to be inappropriate for learning before a good knowledge and performance on ESD techniques. Our aim was to evaluate the feasibility to overcome the learning curve in a western training center of the circumferential en-bloc esophageal endoscopic submucosal dissection in an in vivo animal model and to include this technique in an ESD course at a World Gastroenterology Organisation training center in our hospital.

## 2. Materials and Methods

### 2.1. Ethics

 This work has been carried out in accordance with “The Principles of Humane Experimental Technique, Russel and Burch” [1, 5]. After receiving the approval for the protocol by the ethics and animal research committees at our institution (GAS-14-09/09-1) the procedures were conducted on ten canine models of mongrel breed and weight between 18–20 kilograms that had previously passed the quarantine period under general anesthesia proportionated by an expert veterinarian. During the quarantine period the canine models received adequate care, nutrition, and vaccines by expert veterinarians. The canine models were proportionated to our hospital by the antirabic center following all the stipulated legal procedures in our country. All the procedures were done at the animal lab surgery room with cardiopulmonary monitoring and artificial ventilation by staff surgery members and a staff member of the Gastroenterology Department trained during 1999–2001 at the Fujigaoka hospital of the Showa U. in Yokohama, Japan. After the ESD was completed in each canine model, they were euthanized by an expert veterinarian with an anesthetic overdose, and surgical resection was done by a staff surgeon of the esophagus and stomach carried out according to “the Principles of Humane Experimental Technique, Russel and Burch” [5]. 

### 2.2. Cutting Devices

#### 2.2.1. Needle Knife

The needle knife KD-10Q-1 (Olympus Optical, Tokyo, Japan) was used to produce coagulation marks in the upper and distal esophageal circumferential artificial lesion that afterwards were used to facilitate introduction of the endoscopy needle for the injection of a solution that elevates the submucosal layer [1, 2]. 

#### 2.2.2. Hook Knife

 The hook-type knife KD-620LR (Olympus Optical, Tokyo, Japan) was used to perform the initial circumferential cut in the upper and distal esophageal circumferential artificial lesion [1, 2] and afterwards by hooking and sweeping off the tissue during all the circumferential en-bloc esophageal ESD procedure in the canine models.

#### 2.2.3. Modified Insulated Tipped Knife 2

 The modified IT2, insulated tipped diathermic knife 2 KD-611L (Olympus Optical, Tokyo, Japan) with the small ceramic ball attached to the tip of the blade functioning as an insulator so that the incision and dissection of the mucosa and submucosa in the vertical approach can be performed safely. Because of these IT2 features we use this knife after circumferential incision to make a tunnel from the oral part through the distal gastroesophageal junction (GEJ) and avoid perforation during the initial attempts [1, 2].

### 2.3. Injection Solution for Mucosal and Submucosal Elevation

We use for all the canine models normal saline solution with diluted epinephrine (1 : 10,000) and indigo carmine [1–3] injected to elevate the lesion and to separate the submucosal layer from the muscular layer as needed during all the steps of the ESD depending on the size of the lesion. Also, this solution was used in case of small bleeding sites during the ESD [1, 2] or when the cushion of the submucosal layer was insufficient for a good dissection.

### 2.4. Electrosurgical HF Generator

We used the new electrosurgical HF-generator ESG-100 (Olympus Optical, Tokyo, Japan) with standardized settings for the coagulation and cutting program to avoid complications [1].

### 2.5. Endoscopic Unit and Video Endoscope

All the procedures were done with an endoscopy unit CV-145 (Olympus Optical, Tokyo, Japan) and a video endoscope GIF-Q1145 (Olympus Optical, Tokyo, Japan).

### 2.6. Procedure

**Figure 1:**
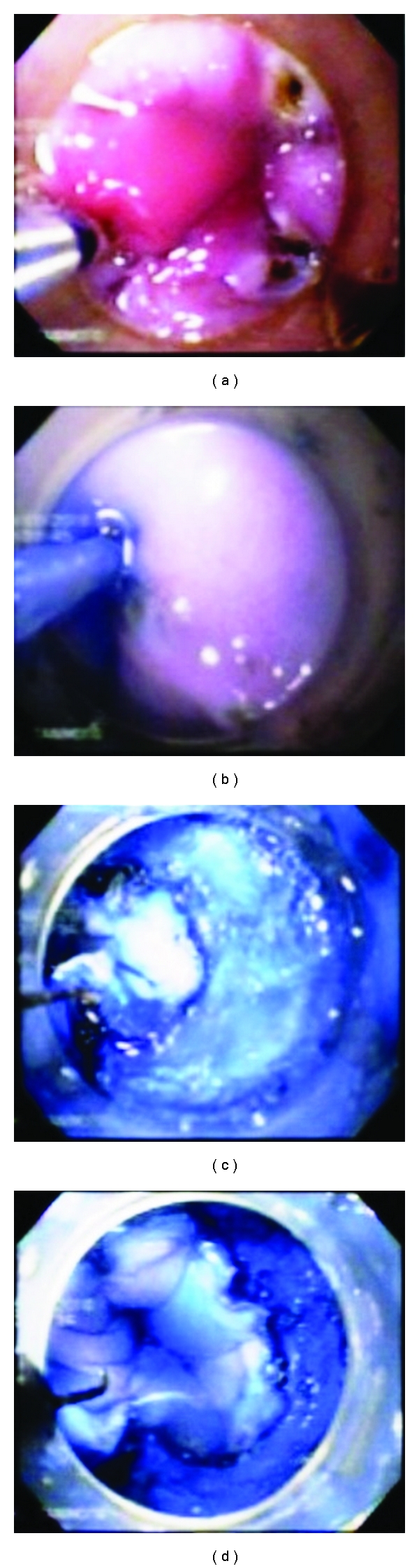
Procedure: (a) artificial lesion marked with coagulation points; (b) injection of normal saline with epinephrine and indigo carmine; (c) and (d) hook knife cutting and sweeping off around the lesion.

**Figure 2:**
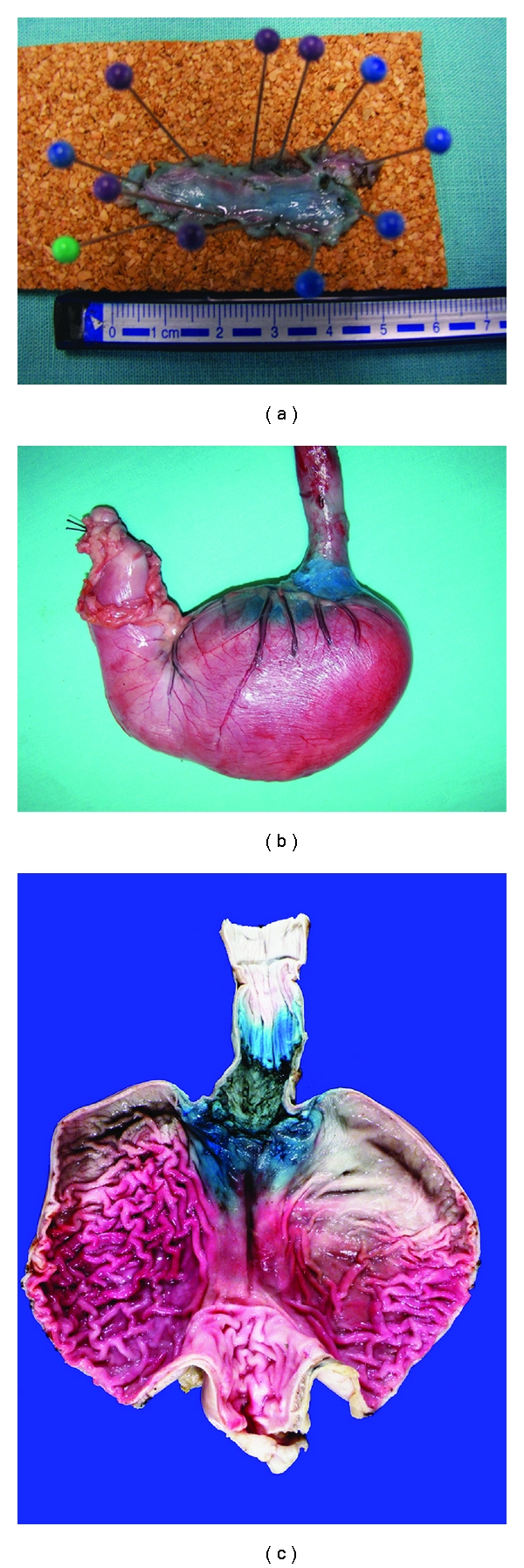
Photographs of (a) the dissected specimen fixed on cork; (b) and (c) the surgical retrieved esophagus and stomach after ESD.

**Figure 3:**
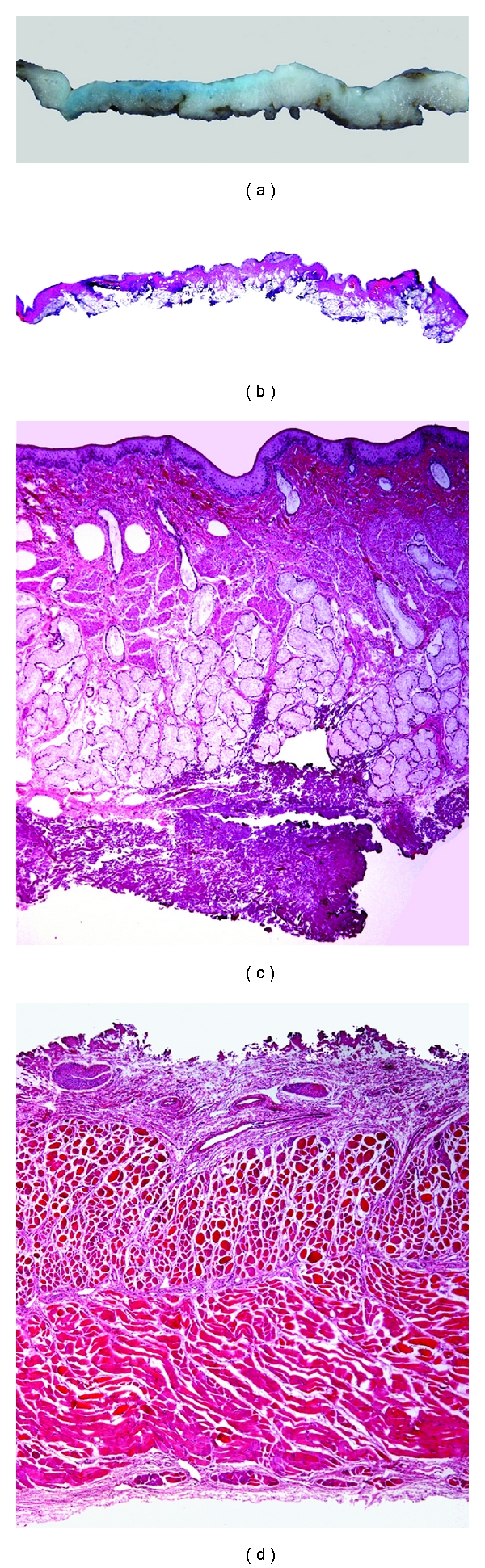
ESD specimens: (a) and (b) macroscopic and microscopic views of the resected specimen; (c) microscopic view of the resected mucosal and submucosal layer and (d) microscopic view of the layer beneath the submucosal dissection demonstrating with the muscular integrity that there were no perforations.

The initial step was to produce by coagulation marks a circumferential artificial lesion at the gastroesophageal junction (GEJ) and above 3–5 cm approx. at the oral site in 10 canine models (one at a time) (Figure 1(a)). The settings for marking the circumferential artificial lesion with the needle knife KD-10Q-1 (Olympus Optical, Tokyo, Japan) was initially powered at “20 watts” with “force coag1” mode, and finally we standardized this parameter to improve efficacy at power “30 watts” with “force coag2” mode also using a soft cap D-201-11804 attached to the tip of the endoscope acting as the surgeons left hand and also for a more precise control of the knifes. After the markings were done at the GEJ and the oral site of the artificial lesion, we used the same coagulation markings to facilitate the introduction of the endoscopy needle tip and inject 2 mL of the lifting solution at each point within the markings, when the conventional needle loose sharp and was unable to penetrate and infiltrate the sub mucosa. It was very important to produce enough elevation of the mucosa with the needle tip securely placed in the submucosal layer to facilitate the tunneling dissection up to the GEJ circumferential incision (Figure 1(b)). This technique also allowed us to perform a one-intention clockwise circumferential clean cut by following the blue stained markings. The tip of the hook knife was rotated as needed by the assistant in order to put the tip of the knife in an appropriate cutting direction (Figures 1(c) and 1(d)). With pulse pedal activations of the Hook knife powered initially at “80 watts” in “pulse cut slow” mode, which was standardized afterwards at power “30 watts” in “pulse cut slow” mode to avoid transmural burns and perforation. We then orientate the dissection plane between the blue stained submucosal layer with the indigo contained in the lifting solution and the whitish red muscle layer at the bottom. We preferred to cut against the upper midpart of the sub mucosa with the distal knife right angled tip in the same direction of the cut and never downwards or without a good vision of the cutting direction, by hooking and pulling at the distal part of the incision and leaving a light blue layer over the muscle (Figures 1(c) and 1(d)). Initially, without enough proficiency in the procedure after the proximal and distal circumferential cuts were done with the Hook knife in the first 3 animal models we then used the IT-knife2 intending to avoid perforation by push and cut in a vertical approach beneath the esophageal sub mucosa layer. Once we had acquired more proficiency in the technique we then continue only with the Hook knife from the beginning through the end of the procedure. After the en-bloc circumferential ESD was completed the grasping forceps FG-49L-1 (Olympus Optical, Tokyo, Japan) were used to retrieve the ESD specimens in each canine model. Afterwards, they were euthanized and surgical resection of the esophagus and stomach was carried out according to “the Principles of Humane Experimental Technique, Russel and Burch” [5]. The ESD specimen, were fixed with needles on a piece of cork and measured then submerged in a formalin solution together with the resected esophagus and stomach, (Figures 2(a), 2(b), and 2(c)), then delivered to the Pathology Department for their study (Figures 3(a), 3(b), 3(c), and 3(d)).

## 3. Results

En-bloc esophageal circumferential ESD was completed without complications in the last 3/10 animal models after 7/10 attempts with perforation. Mean duration for the procedures was 192 ± 35 minutes (range 140–235 minutes). Average size of the retrieved specimens was 16.1 ± 1.19 mm in length (range 15–18 mm) and 51 ± 6.99 in width (range 40–60 mm). Results before and after overcome the learning curve for the first 7/10 and the last 3/10 animal models were, respectively: (a) mean specimen length (mm) 15.57 ± .976 and 17.33 ± .577 (*P* = .021); (b) mean specimen width (mm) 50.71 ± 7.31 and 51.66 ± .857 (*P* = .857); (c) mean procedure duration (min) 204.86 ± 33.81 and 164.33 ± 21.36 (*P* = .096).

 There was no perforation in the last 3/10 animal models. This fact was corroborated by an expert pathologist in the macroscopic and histological study of the specimens (Figures 2 and 3). 

 The size of each specimen, the procedural duration, and its complications are shown in Table 1 for the esophagus en-bloc circumferential ESD using a Hook knife. Descriptive statistics of the pilot study are shown in Table 2. Results before and after overcome the learning are shown in Table 3.

## 4. Conclusions

The technique described here has been extensively used in Asian countries clinical trials proving its efficacy and safety. A major advantage of endoscopic submucosal dissection is to recover an en-bloc specimen for a precise histopathology analysis (invasion deepness together with vertical, horizontal, and lateral margins). However, these techniques are useful only after acquiring proficiency in an accurate diagnosis of the early gastrointestinal cancer lesions with the adequate technology and knowledge of early GI cancer classifications (i.e., Vienna and Paris Classifications; Chromoendoscopy and Magnification) [1–3]. It is also very important to use the appropriate technology and devices with standardized parameters (i.e, Hook knife and IT2-knife; chromoendoscopy stains and HF-generator ESG-100 unit). In recent international endoscopy meetings there have been discussions about the need of a formal training program for these techniques in the Western and Latin America [1]. In the Western countries probably just in Germany, France, Australia, and Portugal the ESD technique has a formal training program. However, the experience of these centers when compared to Asian centers is very small and the incorporation of these procedures in a daily clinical basis has been slow due to Government Health regulations and sometimes even due to commercial regulations and insurance coverage. Also, the brave endoscopists that initiate these procedures in the West have acquired most of their expertise on video forums, hands on workshops, and short-term fellowships in Asian countries probably due to language ability and or their work schedules because most of them were already assistant professors at the moment of ESD training. Finally, the knowhow, the procedures routines, and high-risk populations have some differences between the Western countries that perform ESD and they are not as uniform as in the Asian countries where they have been working in consensus and classifications. Not to mention the ESD training in Asia after animal model practice is always supervised during the clinical practice by an ESD expert that stops and changes the control of the procedure to avoid complications. Also, in our opinion the assistant must be also an expert of ESD in order to work together with the endoscopist as if they were a single operator by providing and changing all the instruments and sometimes anticipating to his requests for a successful performance of the procedure. In our study even though the endoscopy assistants were not experts on ESD they were capacitated by the endoscopist and acquired enough proficiency during the study.

 To evaluate the feasibility to incorporate the en-bloc circumferential esophageal ESD technique for the postgraduate endoscopy fellows in canine models, as we previously mentioned at Section 2 all the ESD procedures were done by a staff member of the WGO training center and of the Gastroenterology Department trained during 1999–2001 at the Fujigaoka Hospital of the Showa U. in Yokohama, Japan. There are several solutions for the lesion cushion during ESD, and they are selected according to the experience and preferences of the endoscopist. Isotonic saline solution is always used alone or in combination with glycerin or sodium hyaluronate with or without indigo carmine and epinephrine. For a long-lasting elevation of the mucosa it is preferred glycerin or sodium hyaluronate, and epinephrine could be obviated with a depurated technique and good control of the bleeding vessels [1, 2]. As for Indigo carmine some experts do not use it [3]. Initially we have planned to use a combination of knifes in order to complete safer and faster the esophageal circumferential ESD first by using Hook knife for the upper and lower initial circumferential cut and afterwards using the IT2-knife to push and cut for tunneling without perforation due to the isolated tip of this knife. However, during the learning curve we became proficient with the use of the Hook knife [6–9] and were able to perform the complete procedure only with this knife as you can see in Table 1. On a case-by-case endoscopy strategy approach a combination of ESD knifes is more convenient, for example, when fibrosis is found due to multiple previous biopsies or because of tumor recurrence after ESD. Also, the small caliber transparent tip cap allows us to keep a good vision of the cutting direction specially when tunneling beneath the lesion acting as the surgeon left hand during the submucosal dissection. ESD is known to be a very long procedure, and its duration depends on case-by-case size and location of the lesion [10–12]. In our study, the mean duration of the ESD procedures was 193 min. However, in the last 3 cases it was shorter and without complications when compared to the former 7 cases. Even when in our study the number of cases is very small with a high complication rate we demonstrate how many cases are necessary prior to obtaining and adequating proficiency on this difficult and high skill procedure in order to avoid the most dangerous complication such as perforation. Advanced proficiency with esophageal ESD offers the possibility of shortening this duration and may help reduce complication rates such as perforation [6–22]. Since location, size, and shape of the neoplastic lesions are not uniform for an optimal resection position at ESD and to deal with the vertical and horizontal approach there is an intense and active research to improve ESD devices that eventually may allow an easier, faster, and safer approach. Some examples are the sophisticated multipurpose scopes and combinations of endoscopy techniques with laparoscopic surgical instruments described in video forums and journals. It is harder to make a cost/effectiveness analysis and discussion for the ESD techniques due to the introduction of these sophisticated technologies. Because ESD performed with a regular single working-channel endoscope requires high skills acquired by tutorial training, there are few training centers around the world in which an endoscopy fellow can be trained in the ESD technique; in consequence it is not possible to incorporate this procedure to the medical practice in the Western and Latin American countries [1, 2]. Also, there is a strong demand for structured ESD training courses because an increasing number of esophageal adenocarcinoma cases and the neccesity of a less invasive therapy that could provide a better quality of life when compared with radical or partial esophageal surgery. A close collaboration between Western and Asian centers is recommended for improvement of the ESD technique and its clinical application [1, 2]. 

The implementation of an ESD course at a World Gastroenterology Organisation western training center will not only help to open numerous research areas that could contribute to the treatment of early GI cancer lesions, but will also help the worldwide dissemination of these techniques. 

Further studies are necessary with a greater number of cases and to analyze the management of post-ESD complications and survival. In our analysis after 7 procedures the endoscopist acquires the proficiency to complete the en-bloc circumferential ESD without perforations in the last 3/10 cases. Although as we mention above this technique is more challenging and considered to be inappropriate for learning before a good knowledge and performance on ESD techniques. However, the final goal to perform ESD is not to resect the lesion in an en bloc fashion, but to save the patient from esophageal cancer related death.

## Figures and Tables

**Table 1:** Circumferential ESD in the esophagus using Hook knife (KD-620LR).

Size of specimen (mm)	Procedural duration (min)	Complications
(1) 15 mm × 40 mm	235 min	Perforation
(2) 17 mm × 45 mm	232 min	Perforation
(3) 15 mm × 50 mm	233 min	Perforation
(4) 15 mm × 60 mm	215 min	Perforation
(5) 17 mm × 50 mm	200 min	Perforation
(6) 15 mm × 60 mm	169 min	Perforation
(7) 15 mm × 50 mm	150 min	Perforation
(8) 17 mm × 45 mm	180 min	None
(9) 17 mm × 50 mm	173 min	None
(10) 18 mm × 60 mm	140 min	None

**Table 2:** Descriptive statistics of circumferential ESD in the esophagus using Hook knife (KD-620LR).

Values	Procedural duration (min)	Specimen length (mm)	Specimen width (mm)
*n* = 10			
mean	192.70	16.1	51
SD	35.30	1.19	6.99
Range	140–235	15–18	40–60

**Table 3:** Results before and after learning curve of circumferential ESD in the esophagus using Hook knife (KD-620LR).

	First 7 cases	Last 3 cases	*P*
Mean specimen lenght (mm)	15.57 (±.976)	17.33 (±.577)	.021
Mean specimen width (mm)	50.71 (±7.31)	51.66 (±7.63)	.857
Mean procedure duration (min)	204.86 (±33.81)	164.33 (±21.36)	.096
Complications	Perforation (7)	None	—
